# Multiple sgRNAs facilitate base editing-mediated i-stop to induce complete and precise gene disruption

**DOI:** 10.1007/s13238-019-0611-6

**Published:** 2019-02-14

**Authors:** Kun Jia, Zongyang Lu, Fei Zhou, Zhiqi Xiong, Rui Zhang, Zhiwei Liu, Yu’e Ma, Lei He, Cong Li, Zhen Zhu, Dejing Pan, Zhengxing Lian

**Affiliations:** 1 Beijing Key Laboratory of Animal Genetic Improvement, China Agricultural University, Beijing, 100094 China; 2 Cambridge-Suda Genomic Resource Center and Jiangsu Key Laboratory of Neuropsychiatric Diseases Research, Soochow University, Suzhou, 215123 China; 3 School of Life Science and Technology, ShanghaiTech University, Shanghai, 201210 China; 4 Key Laboratory of MEMS of Ministry of Education, Southeast University, Nanjing, 210096 China

**Dear Editor,**

Gene editing is a process to introduce desired changes into targeted loci of genomic DNA. Recently, type II clustered regularly interspaced short palindromic repeats-associated Cas9 endonuclease (CRISPR/Cas9) system has been demonstrated as a versatile tool for engineering eukaryote genome (Hsu et al., 2014), such as in mice (Zuo et al., 2017). CRISPR/Cas9-mediated genome editing is achieved by the error-prone DNA repair of non-homologous end joining (NHEJ) after double strand DNA cleavage. However, the editing results are unreliable due to uncontrolled random indels. Moreover, it was also occasionally reported that Cas9 may induce troublesome off-target effects (Hsu et al., 2013; Pattanayak et al., 2013; Cho et al., 2014).

Scientists are making continuous efforts to modify and optimize the gene editing tools. In a landmark study, Komor et al. developed a ‘DNA’ base editor (BE), a novel genome editing tool which is applicable to change C/G base pairs to A/T without introducing DNA double strand breaks. Thereafter, various modifications have been created to the base editor system to improve its editing efficiency. BE3 can introduce C-to-T nucleotide substitution at the window of position 4–8 bases of the non-binding strand of the sgRNA (Komor et al., 2016). BE4max increased efficiency in a variety of mammalian cell types (Koblan et al., 2018). Interestingly, BE3 has been used to introduce early stop codon (TGA, TAG, TAA) from codons (CAA, CAG, CGA, TGG) to terminate gene expression (Kuscu et al., 2017), and provides a safer and much more precise knockout strategy than Cas9-mediated NHEJ (Kim et al., 2017; Komor et al., 2017).

In this study, we used BE3 and BE4max to edit mouse genome by introducing stop codon (i-stop) in coding region of specific genes. We tested a multiple sgRNAs strategy and the results indicated that multiple sgRNAs dramatically increase the efficiencies of BE3-mediated and BE4max-mediated editing in mouse embryos and successfully generated DKO (double knockout) mice by BE3-mediated i-stop targeting *Tyr* and *Pdcd1*.

First, mouse-derived Neuro-2a (N2a) cells were used as testing system. We designed and screened 14 sgRNAs targeting *Tyr* and *Pdcd1* (7 sgRNAs for each gene), respectively. *TYR* gene encodes the tyrosinase enzyme, and its mutations result in impaired tyrosinase production leading to albinism (Witkop, 1979). *PDCD1* is an immune checkpoint gene which guards against autoimmunity and regulatory T cells (Fife and Pauken, 2011).

To screen the candidate sgRNA, BE3 plasmid and individual sgRNA were co-transfected into N2a cells. The results of chromatograms showed, 3 out of 7 sgRNAs for *Tyr* (Tyr-sg1, Tyr-sg2 and Tyr-sg7 targeting exon1) and 3 out of 7 sgRNAs for *Pdcd1* (Pdcd1-sg1, Pdcd1-sg2 and Pdcd1-sg3, targeting exon 1, exon 2 and exon 3, respectively) worked well in modifying the genome in coding regions (Fig. S1). We then test the editing efficiency of BE4max with these six sgRNAs (Fig. S3A). As expected, subsequent TA clone sequencing confirmed that all six sgRNAs introduced stop codon at the predicted sites with BE3 (Fig. S1D) or BE4max (Fig. S3B**)**. For BE3, Tyr-sg1 and Tyr-sg2, Tyr-sg7 generated stop codon (Q48stop, W272stop, W12stop) at the frequencies of 13.3%, 22.2% and 14.3% respectively. On the other hand, Pdcd1-sg1, Pdcd1-sg2 and Pdcd1-sg3 generated stop codon (Q79stop, Q167stop and W12stop) at the frequencies of 10%, 37.5% and 20%. For BE4max, Tyr-sg1, Tyr-sg2 and Tyr-sg7 generated stop codon at the frequencies of 50%, 33.3% and 33.3%, respectively, while Pdcd1-sg1, Pdcd1-sg2 and Pdcd1-sg3 generated stop codon at the frequencies of 30%, 50% and 33.2%. Thus, Tyr-sg1, 2, 7 and Pdcd1-sg1, 2, 3 were selected for the further study.

We then attempted to test the efficiency of i-stop conversion in mouse embryos. To test multiple sgRNAs strategies, different combinations of sgRNAs and BE mRNA or BE4max mRNA were co-injected into zygotes (50 ng/μL BE mRNA and 25 ng/μL sgRNAs) (Fig. 1B).

**Figure 1:**
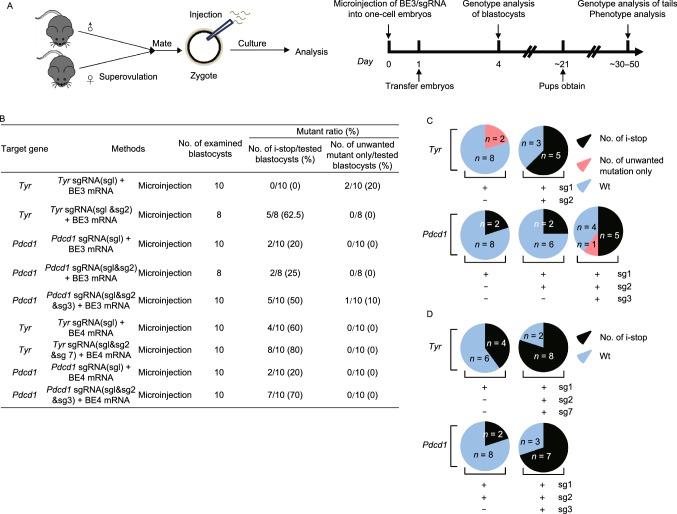

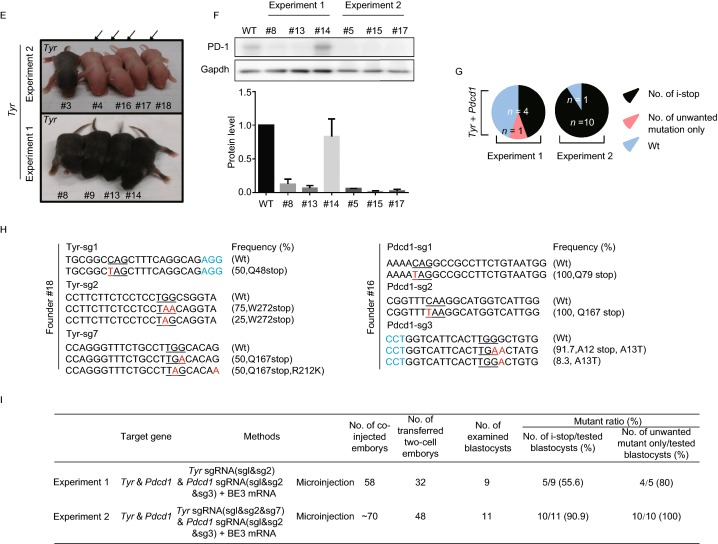
**Efficient C-to-T substitution at Tyr and Pdcd1 loci in mouse embryos and mutant mice**. (A) Representative schematic and timeline of experimental design. After mating and superovulation of mice, sgRNA and BE3 mRNA or BE4max mRNA were co-injected into one-cell embryos, then editing efficiency were detected at blastocyst stage and founder mice. (B) Summary of embryo manipulation. (C) The percentage of different mutation types in mouse embryos by BE3-mediated base editing. Black represents percentage of blastocysts harboring generated stop codons; Red represents percentage of blastocysts harboring unwanted mutations only; Blue represents percentage of Wt (wild type) blastocysts. The number was indicated on the chart. (D) The percentage of different mutation types in mouse embryos by BE4max-mediated base editing. Black represents percentage of blastocysts harboring generated stop codons; Blue represents percentage of Wt (wild type) blastocysts. The number was indicated on the chart. (E) Tyr mutant newborn pups that developed after co-injecting the BE3 mRNA and sgRNA exhibited albino phenotype in their eyes and skin (black arrows, #4, #16, #17 and #18). (F) Representative results of phenotypes of mice from Pdcd1 targeting. Western blot (WB) showing that knockout of Pdcd1 leads to a decrease in PD-1 protein of #5, #8, #13, #15 and #17. (G) The percentage of different mutation types in pups. Black represents percentage of blastocysts harboring induced stop codons; Red represents percentage of blastocysts harboring unwanted mutations only; Blue represents percentage of wild type blastocysts. The number was indicated on the chart. (H) Representative alignments of modified sequences from newborn pups (#16 and #18) using microinjection of BE3 mRNA and sgRNAs into one-stage embryos. The PAM sequences and substitutions are highlighted in blue and red, respectively; The expectedly edited codons are underlined. (I) Summary of the numbers of embryos used and mutants targeting the Tyr and Pdcd1 sites

We successfully introduced stop codon (i-stop) with BE3. For *Tyr*, 2 out of 10 (20%) blastocysts (#5 and #9) for Tyr-sg1 harbored genomic modification of synonymous mutation (G47G), while 5 out of 8 (62.5%) blastocysts (#1–3, #6 and #7) harbored i-stop mutation for Tyr-sg1 combined with Tyr-sg2, indicating that multiple sgRNAs can enhance i-stop introduction in embryos (Figs. 1C and S4A). For *Pdcd1*, 2 out of 10 (20%) (#5 and #6), 2 out of 8 (25%) (#1 and #7) and 5 out of 10 (50%) (#1–3, #7 and #8) blastocysts carried induced stop codon for Pdcd1-sg1, Pdcd1-sg1+2 and Pdcd1-sg1+2+3, respectively (Fig. 1C). It is notable that no indel was observed among all tested blastocysts (Fig. S4A). Only one blastocyst (#5) harbored unwanted mutations (W12C, A13T and V14M) for Pdcd1-sg1+2+3 (Figs. 1C and S4A).

Our further study with BE4max showed more outstanding editing efficiency. For *Tyr*, 4 out of 10 (40%) blastocysts (#1, #4, #6 and #9) for Tyr-sg1 harbored i-stop mutation, while 8 out of 10 (80%) blastocysts (#3–10) harbored i-stop mutation for Tyr-sg1+2+7 (Figs. 1D and S3E). For *Pdcd1*, 2 out of 10 blastocysts (20%) (#3 and #8), 7 out of 10 blastocysts (70%) (#2–5 and #7–9) carried induced stop codon for Pdcd1-sg1 and Pdcd1-sg1+2+3, respectively (Figs. 1D and S3D). Only one embryo (#6) harbored 13 bp deletion at *Tyr* locus. These results further demonstrated the universaltiy of multiple sgRNAs strategy to facilitate efficient i-stop generation in mouse embryos.

With those successes *in vitro*, we assess whether our multiple sgRNAs strategy could achieve complete gene disruption though i-stop conversion *in vivo*. Consistent with previous experimental conditions, BE3 mRNA (total 50 ng/µL) and sgRNAs (Experiment 1: Tyr-sg1+2, Pdcd1-sg1+2+3, total 25 ng/µL) were co-injected into one-cell embryos to target *Tyr* and *Pdcd1* simultaneously, and a total of 9 pups (Founder #6–14) were obtained (Fig. 1I). The results of editing frequencies in tail DNA showed, 5 out of 9 (55.6%) mice were identified carrying genome modification at *Tyr* or *Pdcd1* loci. Among these 5 mice, 4 mice (Founder #8, #9, #13 and #14) harbored i-stop conversion at *Tyr* or *Pdcd1* loci and the last one (Founder #11) harbored A13T conversion only (frequency of 28.6%) for Pdcd1-sg3. Further analysis of these mice indicated that Founder #8 and #9 harbored i-stop conversion for Tyr-sg1 at the frequencies of 33%. Meanwhile, 3 founder mice (#8, #13 and #14) harbored i-stop conversion at *Pdcd1* with the frequencies ranging from 28.6% to 50% (Fig. S4B).

To increase the efficiency of BE3-mediated i-stop, we tried new sgRNA combinations (Experiment 2: Tyr-sg1+2+7, Pdcd1-sg1+2+3). The results showed, 10 out of 11 (90.9%) pups harbored genome modification (Figs. 1I and S4C). Among these 10 mice, 8 (Founder #1, #3, #4, #15–18 and #20) and 6 (Founder #5, #15–19) mice harbored i-stop conversion at *Tyr* and *Pdcd1*, respectively, with the frequencies ranging from 25% to 100%. It is worth notifying that, only 2 out of 20 pups (Founder #5, #9) harbored 5 bp and 2 bp deletion at *Pdcd1* locus, respectively, indicating BE3 is much more precise than wild type Cas9 (Fig. S4C).

Although founder #8 and #9 both harbored W272stop conversion at *Tyr* locus, none of the nine newborns (Experiment 1) displayed the albino phenotype of white skin indicating incomplete gene disruption (Fig. 1E). For those pups from BE3 combined with Tyr-sg1+2+7 and Pdcd1-sg1+2+3, 8 out of 11 newborns (Founder #1, #2, #4, #5, #15–18) displayed Tyr-deficient mice phenotype indicating the increased *Tyr* gene disruption efficiency mediated by multiple sgRNA i-stop strategy. Interestingly, all of the phenotypic mice show white skin over whole body instead of black-and-white skin, the mosaic phenotype which displayed by mice harboring mosaic gene disruption of *Tyr* as previous reported (Zuo et al., 2017). These data further suggest multiple sgRNAs facilitated i-stop conversion and gene disruption, which allows phenotype analysis of founder animals.

To our knowledge, PD-1 protein is encoded by the *Pdcd1* gene and highly expressed in thymus (Yue et al., 2014). To further analyze PD-1 disruption, thymus tissues were isolated by autopsy from 6 mice (#5, #8, #13, #14, #15 and #17). The results showed, Founder #5 and #15 harbored W12stop and Q79Stop conversion at the frequencies of 61% and 100%, respectively. Meanwhile, W12stop conversion for Pdcd1-sg2 and Q167stop conversion for Pdcd1-sg3 were observed in Founder #17 at the frequencies of 90% and 100%, respectively. Founder #8 and #13 harbored W12stop conversion at the frequencies of 49.5% and 50%, respectively. Founder #14 harbored Q167stop conversion at the lower frequency of 30% (Fig. S4C). As expected, low PD-1 level was detected in *Pdcd1* disruption mice (#5, #8, #13, #15 and #17). Among 6 tested mice, harboring high frequencies of i-stop were detected with significant reduction of PD-1 expression to 6%, 12.3%, 6.6%, 1.5% and 2.2%, respectively (Fig. 1F). Founder #14 harboring 30% Q167stop conversion was detected with normal PD-1 expression, which may be explained by the mosaicism. In addition, in total 20 newborns (9 plus 11), 5 mice (Founder #8, #15, #16, #17 and #18) harbored i-stop conversion at both *Tyr* and *Pdcd1*, which demonstrated our strategy can simultaneously disrupt multiple genes *in vivo.*

We evaluated the on-/off-target effect though PCR-based deep sequencing. Using online tool (http://www.rgenome.net/cas-offinder/), we first selected 5 off-target sites for each sgRNA (Table S5). Off-target and on-target sites of six sgRNAs in this study were sequenced using tail DNA from four mice (#4, #16, #17 and #18), and 2 or 3 mice were analyzed for every sgRNA. Based on the sequencing results, no base substitution was detected at any off-target sites (30 sites in total) (Fig. 2C). To further explore the precision of BE3-mediated base editing, a WGS was performed using genomic DNA from two mutant mice (#16 and #18) and a wild-type mouse as the control at depth of about 24×. We analyzed a total of 7,234 sites, including 1 on-target site and 1,069, 2,414, 1,919, 175, 961, 690 off-target sites (with up to 3-nucleatide mismatch) on Tyr-sg1, Tyr-sg2, Tyr-sg7, Pdcd1-sg1, Pdcd1-sg2 and Pdcd1-sg3, respectively (Figs. 2E and S5G). Only the C-to-T substitution within the target window was observed (Fig. 2F).

**Figure 2:**
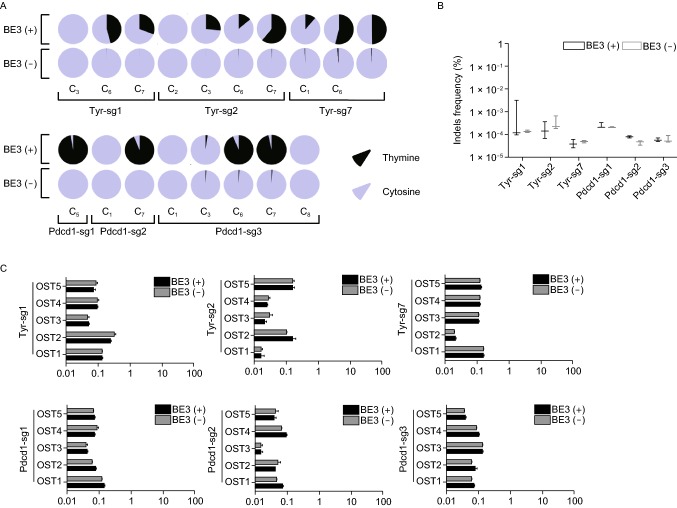

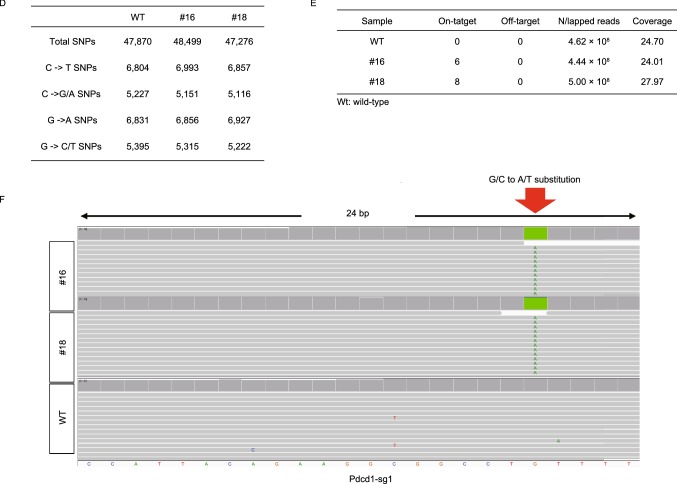
**Comprehensively on- and off-target analysis using targeted deep sequencing and WGS**. (A) The editing frequency of thymine and cytosine were plotted. Black represents thymine and gray represents cytosine. The positions of edited Cs in the Tyr-sg1, Tyr-sg2, Tyr-sg7, Pdcd1-sg1, Pdcd1-sg2 and Pdcd1-sg3 target regions were indicated with the base distal from the PAM set as position 1. (B) The frequencies of desired C-to-T editing to unwanted indels. Statistical analyses show no significant differences between BE3 (+) (black) and BE3 (−) (gray) in indels. (C) Off-target analysis by PCR amplicon-based deep sequencing. The black column represents BE3 (+) and the gray column represents BE3 (−). (D) Summary of genome sequencing analysis. Two mutant mice (#16 and #18) and a wild-type mouse (WT) were sequenced separately using Illumina Novaseq. A total of 47,870, 48,499 and 47,276 SNPs were identified for Wt, #16 and #18, respectively. (E) Summary of on- and off-target analysis. (F) Confirmation of the on-target mutation by the analysis of whole-genome sequencing. Red arrow indicates the G/C to A/T substitution within 24 bp on-target sequence for Pdcd1-sg1

For on-target sites, the average conversions on targeted Cs were 45.75% (C_5_) and 30.63% (C_6_) for Tyr-sg1, 26.36% (C_3_), 14.06% (C_7_) and 61.03% (C_8_) for Tyr-sg2, and 15.05% (C_1_), 53.38% (C_6_), 41.27% (C_7_) for Tyr-sg3. Among these sgRNAs, only sgTyr-7 targeted C_1_ locates out of the reported editing window (C_4_–C_8_). Only one substitution was induced by Pdcd1-sg1 and Pdcd1-sg2 at C_5_ (97.44%) and C_7_ (93.79%), respectively. Targeted substitutions at C_3_, C_6_ and C_7_ were observed at the frequencies of 2.48%, 93.67% and 96.55% respectively for Pdcd1-sg3 (Fig. 2A). As expected, no detectable indel was observed at on-target sites (Fig. 2B). Taken together, multiple sgRNAs perform precise BE3 editing.

Continuous modifications are being made to base editor ever since its discovery to improve the efficiency or precision. From BE1 to current BE4, different components were engineered in base editor system, resulting in steady improvement of editing efficiency. Unlike those biostructural modification, a new strategy was utilized in our study to improve editing efficiency. In summary, we utilized multiple sgRNAs to facilitate i-stop generation of two endogenous genes mediated by different base editor (BE3 and BE4max), resulting in efficient and multiple gene disruption. High throughout sequencing analysis showed multiple sgRNAs strategy facilitated editing is precise with minimal off-target effect and indel. Although the concentration of injected materials was not test, typical concentrations of base editor (50 ng/μL) and sgRNA (25 ng/μL) already achieved decent performance in zygote microinjection. Further studies could be carried out to titrate the injection concentrations and refine the protocol. Taken together, multiple sgRNAs is a universal strategy to achieve efficient gene knockout for phenotype analysis.

## Electronic supplementary material

Below is the link to the electronic supplementary material.

Supplementary material 1 (PDF 1741 kb)
