# Supplementary material for: Multiple sgRNAs facilitate base editing-mediated i-stop to induce complete and precise gene disruption
Source: Protein Cell. 2019 Feb 14;10(11):832–9. doi: 10.1007/s13238-019-0611-6 (PMC6834536; doi:10.1007/s13238-019-0611-6)
Supplement: Supplementary file 1 — Supplementary material 1 (PDF 1741 kb) [file 13238_2019_611_MOESM1_ESM.pdf]

Multiple sgRNAs facilitate base editing-mediated i-stop to induce complete and precise gene disruption

SUPPLEMENTARY INFORMATION

Supplementary figure 1. Efficient C-to-T substitution at *Tyr* and *Pdcd1* loci in N2a by BE3-mediated base editing.

Supplementary figure 2. sgRNA-targeting sites at the *Tyr* and *Pdcd1* locus.

Supplementary figure 3. Efficient C-to-T substitution at *Tyr* and *Pdcd1* loci in N2a and mouse embryos by BE4max-mediated base editing.

Supplementary figure 4. The sequencing results of the embryos and mutant mice using BE3-mediated base editing.

Supplementary figure 5. The off-target sites.

Supplementary Table 1: The sequence of sgRNA oligos.

Supplementary Table 2: Genotyping primers.

Supplementary Table 3: Deep sequencing primers.

Materials and methods

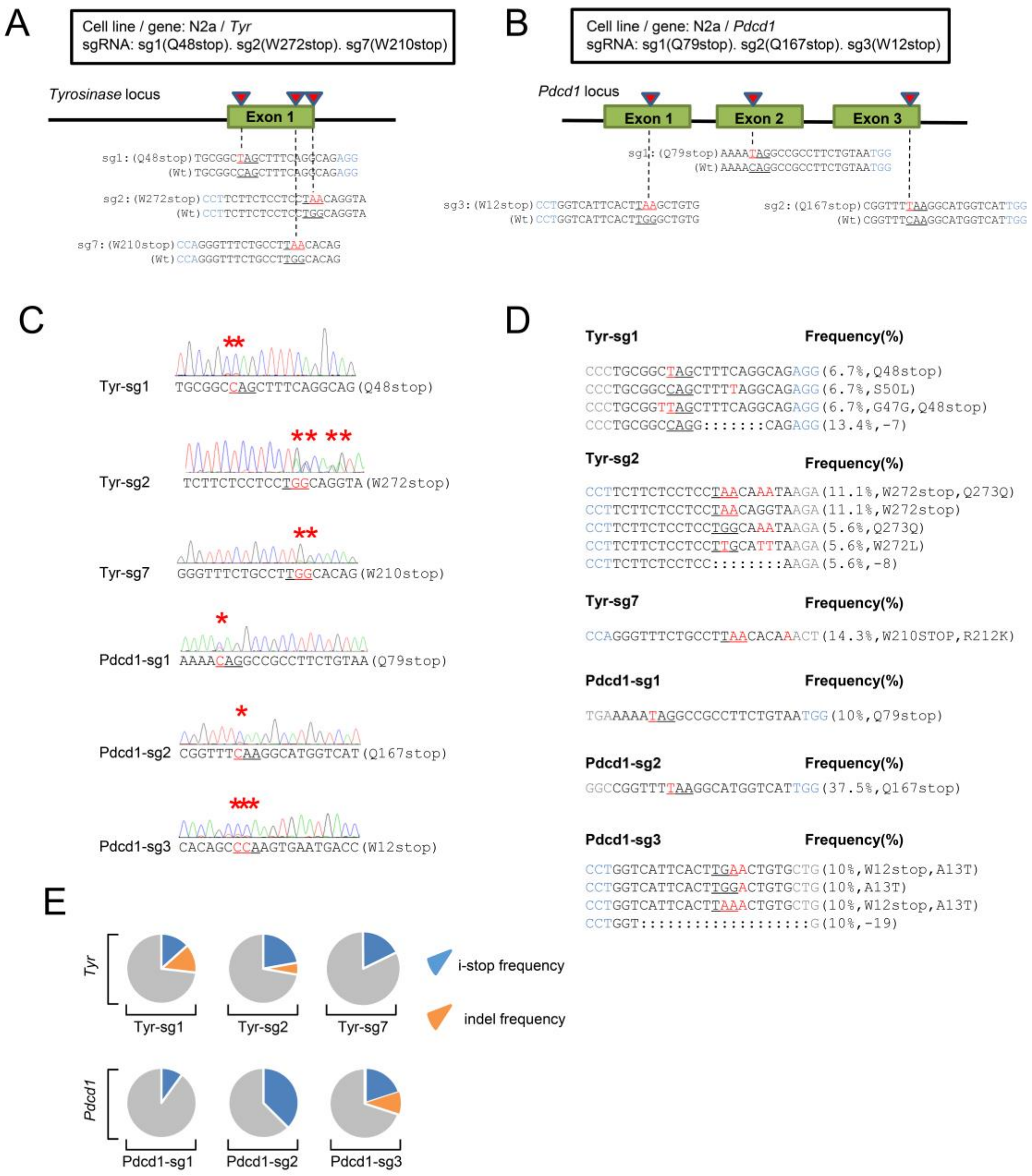

Supplementary figure 1. Efficient C-to-T substitution at *Tyr* and *PD1* loci in N2a by BE3-mediated base editing

(A) Schematic diagram of sgRNA-targeting sites at the *Tyr* locus. Red triangles indicate the predicted substitution sites for the sgRNAs. The PAM sequences and substitutions highted in blue and red, respectively; The expectedly edited codons are underlined. Wt, wild-type.

(B) Schematic diagram of sgRNA-targeting sites at the *Pdcd1* locus. Red triangles indicate the predicted substitution sites for the sgRNAs. The PAM sequences and substitutions highlighted in blue and red, respectively; The expectedly edited codons are underlined.

(C) Sanger sequencing chromatograms of BE3-mediated editing in N2a cells. Red star denotes the substituted nucleotides. The edited codons are underlined and substitutions highlighted in red.

(D) The sequencing results of the N2a using BE3-mediated base editing. The editing efficiencies were detected by sanger sequencing of TA clones for *Tyr* and *Pdcd1* in N2a. Each sample was analyzed at least ten TA clones. The PAM sequences and substitutions are highlighted in blue and red, respectively; The expectedly edited codons are underlined. The column on the right indicates frequencies of mutants and indels.

(E) The analysis of the base editing efficiency of BE3 in N2a co-transfected with different sgRNAs. The efficiencies were detected by Sanger sequencing of TA clones in N2a for *Tyr* and *Pdcd1*. Blue indicates percentage of i-stop mutations; Orange indicates percentage of indels. Each sample was analyzed at least ten TA clones.

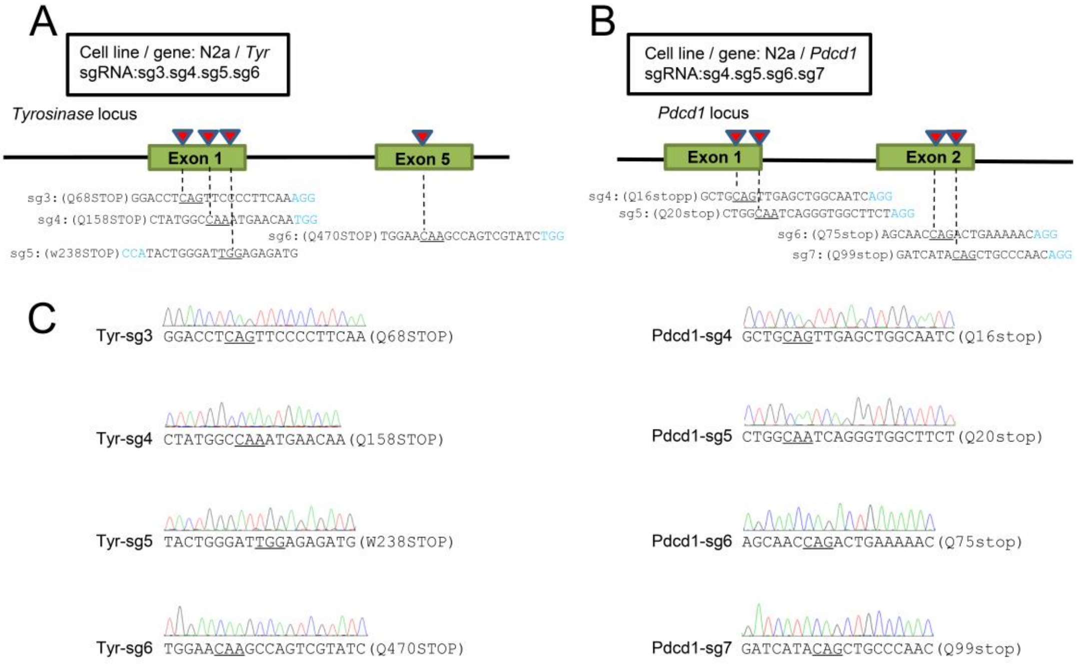

Supplementary figure 2. sgRNA-targeting sites at the *Tyr* and *Pdcd1* loci.

(A) Schematic diagram of sgRNA-targeting sites at the *Tyr* locus. Red triangles indicate the predicted substitution sites for the sgRNAs. The PAM sequences and substitutions are highlighted in blue and red, respectively; The expectedly edited codons are underlined.

(B) Schematic diagram of sgRNA-targeting sites at the *Pdcd1* locus. Red triangles indicate the predicted substitution sites for the sgRNAs. The PAM sequences and substitutions are highlighted in blue and red, respectively; The expectedly edited codons are underlined.

(C) Sanger sequencing chromatogram of BE3-mediated editing in N2a cells. * denotes the substituted nucleotide.

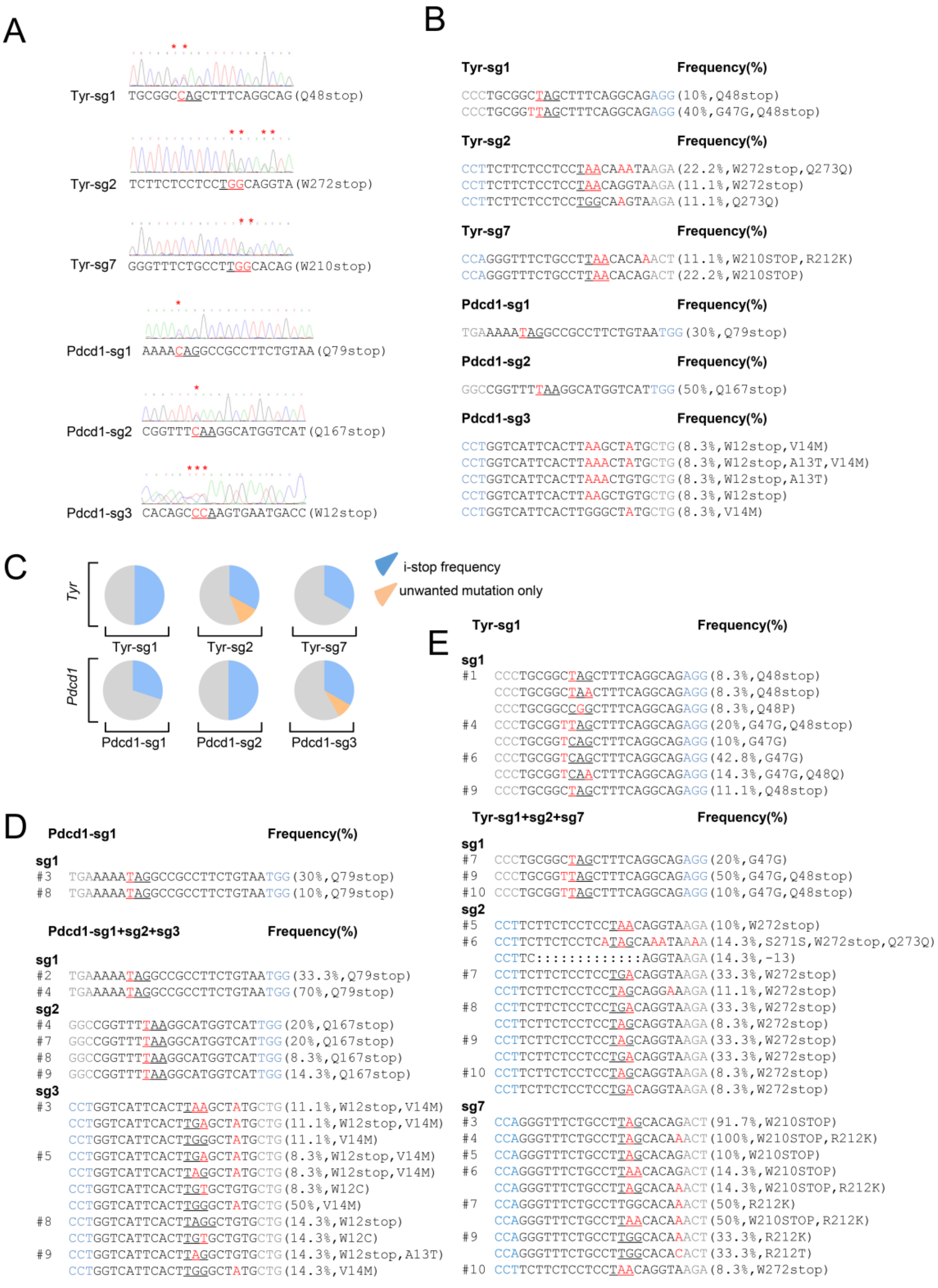

Supplementary figure 3. Efficient C-to-T substitution at *Tyr* and *Pdcd1* loci in N2a and mouse embryos by BE4max-mediated base editing

(A) Sanger sequencing chromatograms of BE4max-mediated editing in N2a cells. Red star denotes the substituted nucleotides. The edited codons are underlined and substitutions highlighted in red.

(B) The sequencing results of the N2a using BE4-mediated base editing. The editing

efficiencies were detected by sanger sequencing of TA clones for *Tyr* and *Pdcd1* in N2a. Each sample was analyzed at least ten TA clones. The PAM sequences and substitutions are highlighted in blue and red, respectively; The expectedly edited codons are underlined. The column on the right indicates frequencies of mutants and indels.

(C) The analysis of the base editing efficiency of BE4max in N2a co-transfected with different sgRNAs. The efficiencies were detected by Sanger sequencing of TA clones in N2a for *Tyr* and *Pdcd1*. Blue indicates percentage of i-stop mutations; Orange indicates percentage of unwanted mutation. Each sample was analyzed at least ten TA clones.

(D) The editing efficiencies were detected by sanger sequencing of TA clones for *Pdcd1* of mouse embryos, each sample was analyzed at least ten TA clones. The PAM sequences and substitutions are highlighted in blue and red, respectively; The expectedly edited codons are underlined. The column on the right indicates frequencies of mutants.

(E) The editing efficiencies were detected by sanger sequencing of TA clones for *Tyr* of mouse embryos, each sample was analyzed at least ten TA clones. The PAM sequences and substitutions are highlighted in blue and red, respectively; The expectedly edited codons are underlined. The column on the right indicates frequencies of mutants.

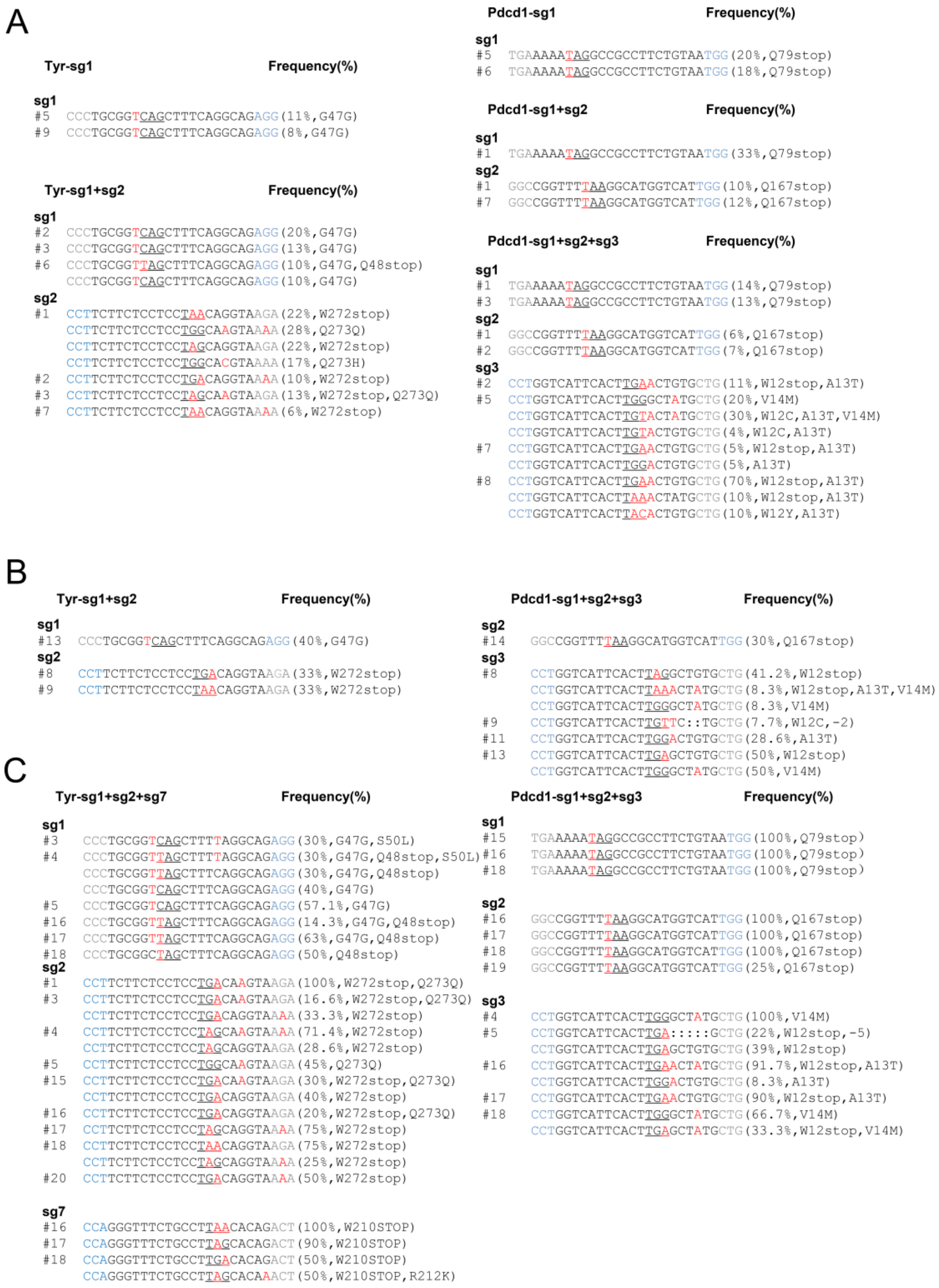

Supplementary figure 4. The sequencing results of the embryos and mutant mice using BE3-mediated base editing.

(A) The editing efficiencies were detected by sanger sequencing of TA clones for *Tyr* and *Pdcd1* of mouse embryos, each sample was analyzed at least ten TA clones. The PAM sequences and substitutions are highlighted in blue and red, respectively; The expectedly

edited codons are underlined. The column on the right indicates frequencies of mutants and indels.

(B) Sanger sequencing chromatograms of DNA from mice co-injecting with sgRNAs (Tyr-sg1+2, Pdcd1-sg1+2+3) and BE3, each sample was analyzed at least ten TA clones. The PAM sequences and substitutions are highlighted in blue and red, respectively; The expectedly edited codons are underlined. The column on the right indicates frequencies of mutants and indels.

(C) Sanger sequencing chromatograms of DNA from mice co-injecting with sgRNAs (Tyr-sg1+2+7, Pdcd1-sg1+2+3) and BE3, each sample was analyzed at least ten TA clones. The PAM sequences and substitutions are highlighted in blue and red, respectively; The expectedly edited codons are underlined. The column on the right indicates frequencies of mutants and indels.

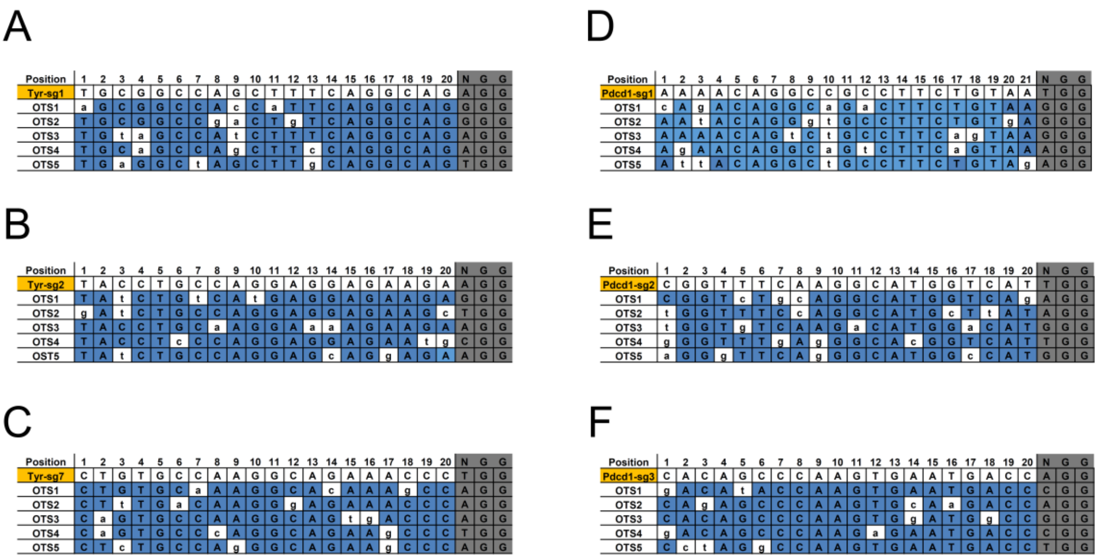

G

sgRNA	potential off-target sequences	Wt	#16	#18	#16 (exclude Wt)	#18 (exclude Wt)
Pdcd1-sg1	175	0	0	0	0	0
Pdcd1-sg2	961	0	0	0	0	0
Pdcd1-sg3	690	0	0	0	0	0
Tyr-sg1	1069	0	0	0	0	0
Tyr-sg2	2414	0	0	0	0	0
Tyr-sg7	1919	0	0	0	0	0

Supplementary figure 5. The off-target sites.

(A) Off-target sites for Tyr-sg1, the lowercase in white cell represents the mismatch nucleotide.

(B) Off-target sites for Tyr-sg2, the lowercase in white cell represents the mismatch nucleotide.

(C) Off-target sites for Tyr-sg7, the lowercase in white cell represents the mismatch nucleotide.

(D) Off-target sites for Pdcd1-sg1, the lowercase in white cell represents the mismatch nucleotide.

(E) Off-target sites for Pdcd1-sg2, the lowercase in white cell represents the mismatch nucleotide.

(F) Off-target sites for Pdcd1-sg3, the lowercase in white cell represents the mismatch nucleotide.

(G) Summary of potential off-target sequences. Up to 2-bp mismatch in seed region and 3-bp mismatch in non-seed region with NRG PAM (using CasOT).

Supplementary Table 1: The sequence of sgRNA oligos

sgRNA	Upstream oligos (5' to 3')	Downstream oligos (5' to 3')
**Tyr-sg1**	ACCGTGCGGCCAGCTTTCAGGCAG	AAACCTGCCTGAAAGCTGGCCGCA
**Tyr-sg2**	ACCGTACCTGCCAGGAGGAGAAGA	AAACTCTTCTCCTCCTGGCAGGTA
**Tyr-sg3**	ACCGGGACCTCAGTTCCCCTTCAA	AAACTTGAAGGGGAACTGAGGTCC
**Tyr-sg4**	ACCGCTATGGCCAAATGAACAA	AAACTTGTTCATTTGGCCATAG
**Tyr-sg5**	ACCGCATCTCTCCAATCCCAGTA	AAACTACTGGGATTGGAGAGATG
**Tyr-sg6**	ACCGTGGAACAAGCCAGTCGTATC	AAACGATACGACTGGCTTGTTCCA
**Tyr-sg7**	ACCGCTGTGCCAAGGCAGAAACCC	AAACGGGTTTCTGCCTTGGCACAG
**Pdcd1-sg1**	ACCGAAAACAGGCCGCCTTCTGTAA	AAACTTACAGAAGGCGGCCTGTTTT
**Pdcd1-sg2**	ACCGCGGTTTCAAGGCATGGTCAT	AAACATGACCATGCCTTGAAACCG
**Pdcd1-sg3**	ACCGCACAGCCCAAGTGAATGACC	AAACGGTCATTCACTTGGGCTGTG
**Pdcd1-sg4**	ACCGGCTGCAGTTGAGCTGGCAATC	AAACGATTGCCAGCTCAACTGCAGC
**Pdcd1-sg5**	ACCGCTGGCAATCAGGGTGGCTTCT	AAACAGAAGCCACCCTGATTGCCAG
**Pdcd1-sg6**	ACCGAGCAACCAGACTGAAAAAC	AAACGTTTTTCAGTCTGGTTGCT
**Pdcd1-sg7**	ACCGGATCATACAGCTGCCCAAC	AAACGTTGGGCAGCTGTATGATC

Supplementary Table 2: Genotyping primers

sgRNA	Forward sequence (5' to 3')	Reverse sequence (5' to 3')
**Tyr-sg1**	CCAGGGGTTGCTGGAAAAGA	GCAGAAACCCTGGTGCTTCA
**Tyr-sg2**	GTTCTTTTCTTACCTCACTTTAGCA	AGGAGGTGCTAACAATACAGTGG
**Tyr-sg3**	CCAGGGGTTGCTGGAAAAGA	GCAGAAACCCTGGTGCTTCA
**Tyr-sg4**	CCAGGGGTTGCTGGAAAAGA	GCAGAAACCCTGGTGCTTCA
**Tyr-sg5**	GTTCTTTTCTTACCTCACTTTAGCA	AGGAGGTGCTAACAATACAGTGG
**Tyr-sg6**	TCAAAAGATCCCAAAAGCAGAGAA	GGAAAACAAAAATGTGCCTCAAGT
**Tyr-sg7**	GTTCTTTTCTTACCTCACTTTAGCA	AGGAGGTGCTAACAATACAGTGG
**Pdcd1-sg1**	CACATTCCTCTCCAGGGGGT	ATGGCAGAGAGCCTAAGAGGT
**Pdcd1-sg2**	GTCCCCAATGGGCCCTG	CATACTTGTTGAGCAGAAGACAGC
**Pdcd1-sg3**	ACTGCTACTGAAGGCGACAC	GCCAGGGATATGAACTCGGG
**Pdcd1-sg4**	ACTGCTACTGAAGGCGACAC	GCCAGGGATATGAACTCGGG
**Pdcd1-sg5**	ACTGCTACTGAAGGCGACAC	GCCAGGGATATGAACTCGGG
**Pdcd1-sg6**	GTCCCCAATGGGCCCTG	CATACTTGTTGAGCAGAAGACAGC
**Pdcd1-sg7**	GTCCCCAATGGGCCCTG	CATACTTGTTGAGCAGAAGACAGC

Supplementary Table 3: Deep sequencing primers

On-/Off-target site	Forward sequence (5' to 3')	Reverse sequence (5' to 3')
**Tyr-sg1-ontarget**	AGCCTGTGCCTCCTCTAAGAA	ACGGTCATCCACCCCTTTGA
**Tyr-sg1-OST1**	TTCCTGTGGAGTTGGCTGGA	CTGCATTTCAAGGGGACACG
**Tyr-sg1-OST2**	TCACTCTGCAGGAGACTTGGG	AGCTTTCAGGACCAAGGACAC
**Tyr-sg1-OST3**	GATGCCTGCTCCAGCATTTG	AGGTACAAGAGGAATGGTGCTG
**Tyr-sg1-OST4**	CCCTTAGAGAGAGCATGGGGC	AGCCAAGCTCAGCTGCTACA
**Tyr-sg1-OST5**	GACCAATGCCCCATAATTGGC	GCTACAGTGCAGACGTGGTA
**Tyr-sg2-ontarget**	GTACTTGGGAGGTCGTCACC	AGAAGAAGGATGCTGGGCTGA
**Tyr-sg2-OST1**	AATGAAGGCCACACCACTCTC	ATTGACCAGGGCAGAGGAGC
**Tyr-sg2-OST2**	CTGCTGCCCTAATTGCACCT	CAGCCGTCCTCCAAAAGCAC
**Tyr-sg2-OST3**	AGTATACATGAGGCCAGGGAG	GACCATGCTGCTGATCTTGTC
**Tyr-sg2-OST4**	TCCTGGGAAGTTAGATAGCTCCA	AGCCAGCCAAGTAAGAGCAG
**Tyr-sg7-ontarget**	TCAAGGGACACACTGCTTGG	CTGCATCTCTCCAATCCCAGT
**Tyr-sg7-OST1**	TGCTCTGGGCCTTTGGATTT	AAACCTCTGGGGCTTGCATT
**Tyr-sg7-OST2**	GAGGCCGGCGGTTTATCTA	CCCACAGCTCTTCGTGTTGT
**Tyr-sg7-OST3**	GATGAGTCAGCAGAGGGCTG	AAGCAGGAGGAGGGAAAACC
**Tyr-sg7-OST4**	AAAGGAGTATTCCCTCCCAGC	ACACATCAGCACCACAAATGTT
**Tyr-sg7-OST5**	GACACACCACTGACTTTGCC	GAATCCCACAGCTGACAAATGG
**Pdcd1-sg1-ontarget**	TCAGAGGGAGCAAATGCCAC	AGCTGTATGATCTGGAAGCGG
**Pdcd1-sg1-OST1**	AACTTGCTTGCAGGTTCACAT	AAACACTGTTGCGCCTCATC
**Pdcd1-sg1-OST2**	GAAAGTGATGCTTCTCGGCG	TGTCTGTATCAAGTTCAGGGACC
**Pdcd1-sg1-OST3**	TGTATTGTAGACCTGGCTGCTG	CACCCAAGTTGCTTCTAGCCT
**Pdcd1-sg1-OST4**	GGCACTGTCTCGGTGATGAG	GTCGTGAGGCAATACAGGCT
**Pdcd1-sg1-OST5**	GAAGGAAGGTGACCCACAGG	GCAGTTCCCTTCCCAACAGA
**Pdcd1-sg2-ontarget**	ACAAGATATCCCAGCCCCTC	AGCAGGGTATGATGAGCCTT
**Pdcd1-sg2-OST1**	TGTGTGAAAGGAAGGTGGGTA	ACAGTCAGATGTTCGGTGTG
**Pdcd1-sg2-OST2**	AAGATGATGCTGCCCCTCAA	CCACAGCTGGCTTGATGTTTA
**Pdcd1-sg2-OST3**	CTATGTGCCGCTAGCCTCTG	TAGAGCTGAGACCCAACGGA
**Pdcd1-sg2-OST4**	TGGCAACTGCATTAGTGGCT	GAGTTGCCATTGTGTGACCA
**Pdcd1-sg2-OST5**	GAGCCTGCCTAACCAGACAT	GTGCCAAGAAGTAAACGCAGA
**Pdcd1-sg3-ontarget**	GGAGACTGCTACTGAAGGCG	AACCGAGGGTGAACGTTTCT
**Pdcd1-sg3-OST1**	TTGCAGGTTGGGTTTTGGTTT	AGGAAACGAAGGCGATGACA
**Pdcd1-sg3-OST2**	TGGGGGAGATGCTCTTGGATA	GCCTCAGCTGAAAACCGTTC
**Pdcd1-sg3-OST3**	GATCCCACATCCCTAGGAGC	TACAGACAACTCTGCCTGAGC
**Pdcd1-sg3-OST4**	CTGATGCTGTGAGTCAGGGT	ATTGCAACAGTCATGGGCAC
**Pdcd1-sg3-OST5**	AGCTACTGTACTAGGAGGCAAGA	TGGCCTAGGAAGAGACCTGA

Materials and methods

Animal ethics statement

The use and care of animals complied with the guideline of the Institutional Animal Care and Use Committee (IACUC) of CAM-SU Genomic Resource Center (CAM-SU GRC), Suzhou, China. Mice were maintained in a SPF (specific pathogen–free) facility under a 12 h dark-light cycle. C57BL/6J and ICR mouse strains were used as embryo donors and foster mothers, respectively.

Vector construct

SgRNA oligos were annealed into pGL3-sgRNA expression vector with U6 promoter and EGFP from Addgene (#107721). The sequence of sgRNA oligos were listed in Table S1.

Cell culture and transfection

Mouse-derived Neuro-2a (N2a) cells were obtained from ATCC (ATCC, CCL-131) and maintained at 37 ℃ with 5% CO2 inside the Modified Eagle Medium (DMEM) (Hyclone, SH30243.01) containing 10% fetal bovine serum (FBS) and 1% penicillin–streptomycin. The transfection procedure was carried out using Lipofectamine 2000 Reagent (ThermoFisher Scientific, 11668019) according to the manufacturer's instructions. In brief, N2a cells were seeded on Poly-D-lysine (Sigma, P4707) coated 12-well plates (JETBIOFIL, TCP010012), and transfection were performed at approximately 70% density about 12 hours after seeding, 0.5μg sgRNA plasmids and 1μg BE3 plasmids were transfected with 3μl Lipofectamine 2000. The medium was replaced with fresh medium at 6h after transfection, cells were harvested using flow cytometry sorting technology at 3 days after transection. Cells were incubated at 37 ℃ with 5% CO2.

In vitro transcription

The BE3 and BE4max plasmid was linearized by AgeI enzyme (NEB, R3552L), and transcribed *in vitro* using T7 ULTRA (Ambion, AM1345). BE3 mRNA and BE4 mRNA was purified using RNeasy Mini Kit (QIAGEN, 74104). SgRNA oligos were annealed into

pUC57-sgRNA expression vector with T7 promoter (Addgene, 51132). Then the targeted sgRNA was amplified and transcribed using MEGAshorttranscript T7 KIT (Ambion, AM1345). The sgRNAs were purified using MEGAclear Kit (Ambion, AM1908) and recovered by alcohol precipitation.

Microinjection of one-cell embryos and embryo transfer.

Super ovulated female C57BL/6J mice were mated to C57BL/6J males, and zygotes were collected from oviducts at E0.5. BE3 mRNA (50 ng/µl) or BE4max mRNA and sgRNAs (25 ng/µl for total sgRNAs in all experiments) were mixed and injected into the cytoplasm of zygotes with well recognized pronuclei. Injected zygotes were cultured to blastocysts for genomic analysis or transferred at 2-cell stage into the oviduct of pseudopregnant ICR female mice to generate offsprings.

Genomic DNA extraction and genotyping

Genomic DNA was extracted from blastocyst stage embryos and tails of newborn mice, and then subjected to targeted deep sequencing and Sanger sequencing. Embryos genomic DNA was extracted using QuickExtract™ DNA Extraction Solution (Lucigen, QE09050), 10 µl QuickExtract™ DNA Extraction Solution were added to blastocysts that developed normally, following digest was performed according to the manufacture's protocol. Genomic DNA of N2a cells and mouse tails was extracted using phenol-chloroform method. 500 µl Lysis buffer (10 µM Tris-HCl, 0.4 M NaCl, 2 µM EDTA, 1% SDS) supplemented with 5 µl proteinase K (10 mg/mL) (Yeasen, 10401ES60) was added to harvested cells or mouse tails and digest at 55 ℃ overnight. The primers used for genotyping were listed in Table S2.

Western blot

Total proteins were extracted from thymus of mice using RIPA lysate containing 1% Phenylmethanesulfonyl fluoride (PMSF) and 1% Protease Inhibitor Cocktail (PIC). Protein was then quantified using a Bradford assay. Equal amounts of soluble protein were separated by SDS/PAGE and transferred onto a polyvinylidene difluoride membrane

(Roche). Immunoblotting was conducted using antibodies specific for PD-1(1:1,000, R&D Systems) and GAPDH (1: 3,000, Sigma-Aldrich). Variations in sample loading were estimated by densitometry and corrected based on the GAPDH band intensities.

Targeted deep sequencing

Possible off-target sites were predicted from Cas-OFFinder (http://www.rgenome.net/cas-offinder) and were amplified from mouse genomic DNA using Phanta® Max Super-Fidelity DNA Polymerase (Vazyme, P505-d3). The off-target sites were listed in Table S3 and the primers used for targeted deep sequencing were listed in Table S4. Non-specific sequences in the PCR products were eliminated by gel electrophoresis. PCR products with different barcodes were pooled together for deep sequencing using the Illumina HiseqXten (2 × 150) platform at Novogene, Nanjing, China.

Whole-genome sequencing

Genomic DNA was extracted by phenol–chloroform method. Libraries were sequenced using the Novaseq platform (Illumina) at Novogene, Nanjing, China.

Data availability

High-throughput sequencing data has been deposited in the NCBI Sequence Read Archive database under accession code (). Plasmids pGL3-U6-sgRNA-EGFP (#107721) and pCMV-BE3 (#73021) are available from Addgene.
